# Supplementary material for: Co-movement of astral microtubules, organelles and F-actin by dynein and actomyosin forces in frog egg cytoplasm
Source: eLife. 2020 Dec 7;9:e60047. doi: 10.7554/eLife.60047 (PMC7759381; doi:10.7554/eLife.60047)
Supplement: Transparent reporting form [file elife-60047-transrepform.docx]

***eLife’s* transparent reporting form**

We encourage authors to provide detailed information *within their submission* to facilitate the interpretation and replication of experiments. Authors can upload supporting documentation to indicate the use of appropriate reporting guidelines for health-related research (see [EQUATOR Network](http://www.equator-network.org/%20)), life science research (see the [BioSharing Information Resource](https://biosharing.org/)), or the [ARRIVE guidelines](http://www.plosbiology.org/article/info:doi/10.1371/journal.pbio.1000412) for reporting work involving animal research. Where applicable, authors should refer to any relevant reporting standards documents in this form.

If you have any questions, please consult our Journal Policies and/or contact us: [editorial@elifesciences.org](mailto:editorial@elifesciences.org).

**Sample-size estimation**

- You should state whether an appropriate sample size was computed when the study was being designed
- You should state the statistical method of sample size computation and any required assumptions
- If no explicit power analysis was used, you should describe how you decided what sample (replicate) size (number) to use

Please outline where this information can be found within the submission (e.g., sections or figure legends), or explain why this information doesn’t apply to your submission:

- Biological replicates are extracts prepared from different batches of eggs, and technical replicates are experiments with the same extract.
- When comparing different conditions (e.g., control vs dynein inhibited), we used the same extract for all conditions, with experiments often run in parallel or sequentially; then we performed biological replicates of such comparisons.
- All results are based on at least 5 and often >10 technical replicates, across at least 3 biological replicates, a common standard for experiments with freshly prepared *Xenopus* egg extracts. Results were highly reproducible across replicates.
- To support the claim that all cytoplasmic networks moved away from interaction zones, we estimated network velocities independently by particle image velocimetry (PIV), as well as the MTOC velocity by particle tracking, then we compared the velocities (Fig 3). Networks moved away from interaction zones at ~5 µm/min, and velocity differences between networks were ~1 µm/min. As networks moved away from interaction zones ~5x faster than relative movement between them, statistical methods were not required to show that all networks moved away from interaction zones.
- To support the claim that artificial dynein cargoes moved slower with F-actin intact than F-actin fragmented: “With F-actin intact, the anti-HOOK2 beads moved inwards at a constant speed of 0.2 ± 0.1 µm/s throughout asters … When F-actin was fragmented with Cytochalasin D, the anti-HOOK2 beads moved at 0.7 ± 0.2 µm/s …” Page 19, lines 336-338. The mean velocities differed by more than the sum of the standard deviations. Velocity distributions are shown in Figs 8D,H.

**Replicates**

- You should report how often each experiment was performed
- You should include a definition of biological versus technical replication
- The data obtained should be provided and sufficient information should be provided to indicate the number of independent biological and/or technical replicates
- If you encountered any outliers, you should describe how these were handled
- Criteria for exclusion/inclusion of data should be clearly stated
- High-throughput sequence data should be uploaded before submission, with a private link for reviewers provided (these are available from both GEO and ArrayExpress)

Please outline where this information can be found within the submission (e.g., sections or figure legends), or explain why this information doesn’t apply to your submission:

- Aster separation movement in *Xenopus* egg extracts: “This kind of separation movement was observed in hundreds of image sequences …” Page 5, line 106.
- Dynein and actomyosin contributions to aster movement: “These findings were qualitatively confirmed by visual inspection and partial analysis of more than 10 experiments using multiple extracts.” Page 6, lines 138-139.
- Circular oscillation on HOOK2-functionalized coverslips: “This 2D-oscillatory movement was observed in >10 different experiments using different batches of extract …” Page 12, lines 222-223.
- Advection of fluorescein with oscillating asters: “Similar results were obtained in >10 experiments in 3 extracts.” Pages 13-14, lines 246-247.
- Transport of organelles toward MTOCs: “The ER intensity around MTOCs increased to ~2 fold higher than the intensity outside the aster (Fig 6A’) in >5 examples scored.” Page 15, lines 271-272.
- The only result that was not highly reproducible was the burst of organelle movement near the aster surface in control with F-actin intact. To handle the variation, we reported the fraction of experiments in which the burst was observed, showed one example of each in the main text, and discussed factors contributing to the variation: “Out of 11 extract preps, we observed a burst of inward ER movement at the aster periphery in 7 extracts (64%) as in Fig 7C, and observed weaker or no burst in the remaining extracts as in Fig 7F. Factors that seem to lessen the burst of inward movement include higher concentrations of spontaneously nucleated MTs outside the aster, and insufficient passivation of the coverslips.” Page 17, lines 313-317.
- In contrast, the burst of organelle movement was highly reproducible with F-actin fragmented: “When F-actin was fragmented, a burst of organelle transport at the growing aster periphery was observed in all experiments (>10 repeats with different extracts).” Page 17, lines 320-321.

**Statistical reporting**

- Statistical analysis methods should be described and justified
- Raw data should be presented in figures whenever informative to do so (typically when N per group is less than 10)
- For each experiment, you should identify the statistical tests used, exact values of N, definitions of center, methods of multiple test correction, and dispersion and precision measures (e.g., mean, median, SD, SEM, confidence intervals; and, for the major substantive results, a measure of effect size (e.g., Pearson's r, Cohen's d)
- Report exact p-values wherever possible alongside the summary statistics and 95% confidence intervals. These should be reported for all key questions and not only when the p-value is less than 0.05.

Please outline where this information can be found within the submission (e.g., sections or figure legends), or explain why this information doesn’t apply to your submission:

- Raw data are shown in Figs 2, 3, 4, 5, 8.
- Data averaged over aster quadrants are shown in Figs 6, 7.
- Mean and standard deviation of cargo speeds are calculated in Fig 8.

(For large datasets, or papers with a very large number of statistical tests, you may upload a single table file with tests, Ns, etc., with reference to sections in the manuscript.)

**Group allocation**

- Indicate how samples were allocated into experimental groups (in the case of clinical studies, please specify allocation to treatment method); if randomization was used, please also state if restricted randomization was applied
- Indicate if masking was used during group allocation, data collection and/or data analysis

Please outline where this information can be found within the submission (e.g., sections or figure legends), or explain why this information doesn’t apply to your submission:

- Samples were not allocated into experimental groups.

**Additional data files (“source data”)**

- We encourage you to upload relevant additional data files, such as numerical data that are represented as a graph in a figure, or as a summary table
- Where provided, these should be in the most useful format, and they can be uploaded as “Source data” files linked to a main figure or table
- Include model definition files including the full list of parameters used
- Include code used for data analysis (e.g., R, MatLab)
- Avoid stating that data files are “available upon request”

Please indicate the figures or tables for which source data files have been provided:

- The analysis is described in the Methods, and we are uploading the source data files to GitHub https://github.com/jamespelletier/Co-movement
